# Local Translation in Growth Cones and Presynapses, Two Axonal Compartments for Local Neuronal Functions

**DOI:** 10.3390/biom10050668

**Published:** 2020-04-25

**Authors:** Yukio Sasaki

**Affiliations:** Functional Structure Biology Laboratory, Department of Medical Life Science, Yokohama City University Graduate School of Medical Life Science, 1-7-29 Suehiro-cho, Tsurumi-ku, Yokohama 230-0045, Japan; y_sasaki@yokohama-cu.ac.jp

**Keywords:** local translation, growth cone, presynapse, RNA-binding protein, ribonucleoprotein granule, phase separation

## Abstract

During neural development, growth cones, very motile compartments of tips of axons, lead axonal extension to the correct targets. Subsequently, presynapses, another axonal compartment with vigorous trafficking of synaptic vesicles, emerge to form functional synapses with postsynapses. In response to extracellular stimuli, the immediate supply of proteins by local translation within these two axonal compartments far from cell bodies confers high motility of growth cones and active vesicle trafficking in presynapses. Although local translation in growth cones and presynapses occurs at a very low level compared with cell bodies and even dendrites, recent progress in omics and visualization techniques with subcellular fractionation of these compartments has revealed the actual situation of local translation within these two axonal compartments. Here, the increasing evidence for local protein synthesis in growth cones and presynapses for axonal and synaptic functions has been reviewed. Furthermore, the mechanisms regulating local translation in these two compartments and pathophysiological conditions caused by dysregulated local translation are highlighted.

## 1. Introduction

Neurons are the longest type of cell in the human body. Typically, neurons are composed of a cell body (including a nucleus), several highly branched dendrites that receive signals from other neurons, and a single axon that sends signals to target cells; each neuron has 1000–10,000 synapses, which are information centers for processing neuronal signals. In the case of human corticospinal neurons, the length of dendrites is several centimeters, while the length of the axons is about 1 m. In rat hippocampal neurons, proteins that are translated in cell bodies are transported anterogradely at less than 40 cm/day to the peripheral parts of axons, even with a fast transport system [1]. Most cytoskeletal and cytosolic proteins propagate only a few millimeters per day. Considering this highly polarized morphology and relatively slow transport of proteins, neurons have an increased need to use local translation in neurites (axons and dendrites) for immediate responses to changes in the extracellular environment around distal neurites compared with typically shaped cells.

Although axons, which are longer than dendrites, supposedly have more need for a local source of proteins, local translation in dendrites has been more extensively studied because local translation is easier to detect in dendrites than axons (reviewed by [2]). Electron microscopic (EM) analysis revealed the existence of ribosome particles in proximal dendrites of monkey spinal cord neurons in 1965 [3]. In the 1980s, the Steward group identified polysomes, which are clusters of two or more ribosomes with mRNA, beneath the base of dendritic spines of the rat adult and developing dentate gyrus [4,5]. Polysomes increased from 12% to 39% in postsynapses after stimulation to induce neuronal plasticity, also known as long-term potentiation (LTP) [6]. As polysomes indicate actively translating ribosomes, these results indicate that translation in postsynapses was upregulated during LTP. Transcriptome analysis of neuropils in the stratum radiatum and lacunosum moleculare of CA1 in the rat hippocampus revealed 2550 transcripts localized within dendrites and/or axons [7]. Because neuropil mRNAs include a significant number of mRNAs derived from axons, localization of dendrite mRNAs was confirmed using fluorescent in situ hybridization (FISH). FISH analysis identified 74 mRNAs, such as *Camk2a* (encoding calmodulin-dependent protein kinase CaMKIIα) and *Dlg4* (encoding postsynaptic density (PSD) protein 95 (PSD-95)), localized in microtubule associated protein 2 (MAP2)-positive dendrites [7]. The existence of polysomes and specific mRNAs suggest local translation in dendrites. 

**Figure 1:**
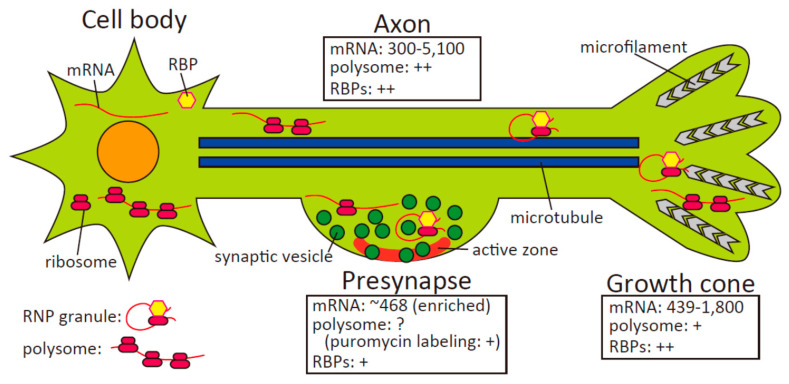
Local translation in axons, growth cones, and presynapses. mRNAs localize in axons, growth cones, and presynapses. Polysomes exist in axons and growth cones, but not in presynapses. However, nascent peptides are metabolically labeled with puromycin, suggesting translation by monosomes at presynapses. RNA-binding proteins (RBPs) localize in axons and growth cones. Recently, the existence of RBPs was also confirmed in presynapses. Transport granules, one type of neuronal ribonucleoprotein (RNP) granule, suppress translation during axonal transport and maintain its suppression in growth cones and presynapses. Once growth cones and presynapses are stimulated by extracellular signals, RNP granules are dispersed and translation is initiated.

The existence of local translation in axons was controversial because of difficulties detecting polysomes in axons of adult mammalian neurons. However, detecting polysomes in axons of developing mammalian neurons was less difficult compared with adult axons. Notably, it was observed that the density of polysomes in axons was only about one-eighth of the density in dendrites of developing rat hippocampal neurons in culture [8]. Early evidence of axonal translation was also provided by invertebrate models. In 1968, metabolic labeling experiments demonstrated that squid giant axons separated from their soma could incorporate radioactive amino acids into newly synthesized proteins [9]. In the 1980s and 1990s, the existence of noncoding ribosomal RNAs [10], mRNAs [11], and polysomes [12] was identified in squid axons, indicating active translation in axons. Electron spectroscopy imaging using isolated myelinated Mauthner goldfish axons revealed the existence of discrete ‘plaque-like’ ribosome-containing structures subjacent to the plasma membrane [13]. Comparable periaxoplasmic ribosomal plaques in close proximity to the plasma membrane were identified in myelinated axons of lumbar spinal nerve roots in rabbits and rats [14], indicating polysome puncta in mammalian axons near the plasma membrane. Furthermore, subsequent studies clarified that growth cones of vertebrate axons contain specific mRNAs, polysomes, and translation initiation factors, which were involved in growth cone responses [15,16,17]. These pioneering studies demonstrated that local translation indeed occurs in axons, against all preconceptions of axons having no ability to translate proteins. Recent progress in omics methods allows analysis of the axonal transcriptome and translatome (the set of all mRNAs bound to the ribosomes) using physically isolated axons, resulting in additional critical evidence for local translation in axons. Because it is difficult to physically isolate dendrites, this is an advantage of analyzing local translation in axons. This review highlights local translation in axons and two axonal compartments, growth cones and presynapses, which provide local neuronal functions such as axon guidance and neurotransmitter release. In particular, recent and rapid progress of local translation research in presynapses is focused upon, and compared with local translation in growth cones. “Local translation” is defined using three criteria: existence of mRNAs, actively translating ribosomes, and regulatory proteins such as RNA-binding proteins (RBPs). Finally, potential roles of local translation in physiological and pathophysiological conditions are discussed (Figure 1). 

## 2. Local Translation in Axons 

Axons have unique features different from dendrites. Indeed, because axons are generally longer than dendrites, axons are physically isolated by special culture systems such as microfluidic chambers and compartmentalized chambers. Axonal compartments of microfluidic chambers are far enough from the cell body compartments (e.g., 150–900 µm) that axonal compartments are free from cell bodies or dendrites, which are shorter than axons [18]. A combination of microfluidic chambers and microarray analysis revealed that >300 mRNA transcripts exist in axons of rat cortical neurons [19] (Figure 1). Gene ontology (GO) analysis revealed that mRNAs related to translation, mitochondria, intracellular transport, and the cytoskeleton are highly enriched in axons of the central nervous system. Using compartmentalized chambers, which separate sensory axons from cell bodies, microarray analysis identified about 2600 (embryonic axons) and 2900 (adult axons) mRNAs enriched for protein synthesis, mitochondrial functions, and neurite-growth related proteins in the axons of rat embryonic and adult sensory dorsal root ganglion neurons [20]. Laser capture microdissection (LCM), a method for isolating specific cells of interest using a laser under a microscope, was applied to isolate axons and growth cones from cell bodies. Using axons of retinal ganglion cells (RGCs) of the African clawed frog *Xenopus* that were microdissected by LCM, microarray analysis revealed an axonal transcriptome composed of transcripts of 5100 genes [21]. Functional analysis showed that protein synthesis and RNA post-transcriptional modification constituted the major functional categories of axonal mRNAs. These results indicate that specific mRNAs are localized in axons for local translation, energy metabolism, and cytoskeletal reorganization. (Comparison of transcriptomes between axons and growth cones will be discussed in Section 3.) The Holt group invented a unique method to identify mRNAs bound to translating ribosomes by affinity purification (TRAP) with RiboTag, which uses a knock-in mouse line expressing an HA-tagged ribosomal protein L22 [22]. TRAP-RiboTag analyzed ribosome-bound mRNAs (the translatome) in distal axons of knock-in mouse RGCs at different developmental stages [22]. The number of mRNAs in the axonal translatome reached a peak (>2000 mRNAs) at postnatal day 0.5 (P0.5), when axon branching and synapse formation are prominent, and then gradually decreased postnatally; whereas, the number of mRNAs in the retinal soma remained almost steady during this period. GO analysis of axonal and somal translatomes revealed the enrichment of mRNAs encoding proteins already known to function in axons and their compartments (e.g., axon, synapse, actin cytoskeleton, and neuronal projection) in the axonal translatome, whereas mRNAs encoding nuclear proteins (e.g., modifiers of chromatin structure) were enriched in the somal translatome [22]. These findings suggest that specific mRNAs related to axonal functions are enriched and translated in axons (Figure 1). 

Axonal mRNAs are translationally regulated. While translation is suppressed during long-distance transport of mRNAs along axons, local translation is promoted at specific sites within axons, such as growth cones and presynapses [23,24] (Figure 1). RBPs possessing sequence specificity for mRNAs are involved in translational regulation of specific mRNAs of the axonal transcriptome [23,24]. RBPs form neuronal ribonucleoprotein (RNP) granules, which are membraneless, macromolecular condensates that include specific sets of mRNAs and RBPs to perform a dual function in regulating translation: suppression of translation and promotion of translation upon activation [24,25,26]. Neuronal RNP granules, which include transport granules, stress granules, and P-bodies, have the diversity to exert a variety of functions [24,25,26]. Transport granules suppress translation during mRNA transport. The motor protein kinesin-5 is associated with RBPs such as Fragile X mental retardation protein (FMRP, a causative gene product of Fragile X syndrome) and fused in sarcoma (FUS), which are related to amyotrophic lateral sclerosis and frontotemporal dementia [27]. Because these RBPs are trafficked in axons as granules [28,29], they are considered to be involved in the suppression of translation during axonal transport. Stress granules, which contain RBPs such as GTPase-activating protein (SH3 domain)-binding protein 1 (G3BP1), TIA-1, and 40S ribosomal subunits, are formed to suppress translation during cellular stresses such as oxidative stress [24,25,26]. P-bodies, which contain RBPs such as Dcp1a (encoding mRNA-decapping enzyme 1A) and GW182, localize to sites of translational repression and/or mRNA degradation [24,25]. RNP granules assemble through liquid–liquid phase separation (LLPS) via protein–protein interactions of RBPs and RNA–RBP interactions [30,31,32]. LLPS concentrates RBPs and RNA by demixing RNP granule components from the cytoplasm, resulting in the suppression of translation. Membraneless RNP granules formed by LLPS enable rapid and reversible assembly/reassembly of the RNA–RBP complex via posttranslational modifications (PTMs), such as phosphorylation and methylation, in response to extracellular stimuli [30,31,32]. This unique property of RNP granules is suitable for regulation of local translation at specific sites of axons, such as growth cones and presynapses. One example is the role of FUS in translation in growth cones. A mutation in FUS, which increases the propensity for phase separation, was found to suppress local translation in growth cones [33,34]. These findings suggest the possibility that granule formation by LLPS suppresses local translation in growth cones and presynapses. Live imaging using endogenous RNA labeled with fluorescent dye (Cy5)-conjugated UTP demonstrated that endogenous RNA formed punctate granules, which moved vigorously in anterograde or retrograde directions or bidirectionally [35,36]. RNA granules containing Cy5 were considered to be transport granules, and fused and split frequently, indicating typical behaviors of condensates formed by LLPS. These granules occasionally entered the growth cones, and some moving granules suddenly stopped at sites of branch emergence of axon arbors. Live imaging using a fluorescent reporter of β-actin translation revealed that de novo local synthesis of β-actin occurs in close proximity to docked RNA granules [35], suggesting the involvement of RNA granules in local translation of β-actin at sites of new branch emergence. Because nascent branches arise at newly formed presynaptic sites [37,38], it is possible that RNA granules are involved in presynapse formation via regulation of local translation. In the following sections, local translation in growth cones and presynapses are discussed in more detail, including types of proteins translated, mechanisms for regulation of local translation, and physiological roles of local translation in these two axonal compartments.

## 3. Local Translation in Growth Cones

Growth cones, which are a very motile and amoeba-like part of axon tips, perceive extracellular cues to determine the direction of extension during development [39,40]. Cytoskeletal proteins and endocytic/exocytic vesicles are highly abundant in growth cones, and cytoskeletal reorganization and vesicle trafficking occur vigorously in actively motile growth cones. EM analysis elucidated the presence of polysomes in growth cones in developing hippocampal neurons [8,15], indicating active translation within growth cones. FISH analysis demonstrated the localization of some specific mRNAs of cytoskeleton proteins, such as β-actin and growth-associated protein 43 (GAP-43), in growth cones [15,41]. A combination of LCM and microarray analyses of RGC axon growth cones in mouse and *Xenopus* revealed growth cone transcriptomes composed of transcripts of 1800 and 439 genes, respectively [21] (Figure 1). Comparing transcripts between microdissected growth cones and axons in *Xenopus* RGCs, 58 mRNAs were more enriched in growth cones than in axons [21]. Among mRNAs enriched in growth cones, the largest functional categories were cytoskeletal-related proteins (23%) and protein synthesis (15%), suggesting that specific mRNAs are sorted to growth cones according to demand. Proteomic analysis also confirmed the enrichment of cytoskeletal-related and translation-related proteins in growth cones [42]. In addition, RNA binding proteins, such as zipcode-binding protein 1 (ZBP1) and heterogeneous nuclear ribonucleoproteins (hnRNPs), account for 1% of the growth cone proteome [42,43]. Localization of specific mRNAs, actively translating ribosomes (polysomes), and RBPs in growth cones indicate local translation of specific proteins in growth cones (Figure 1). 

Growth cones sense axon guidance cues in the extracellular environment and navigate axon extensions to find their correct targets [44,45]. Accumulating evidence suggests that local translation plays a critical role in growth cone turning in response to axon guidance cues [46,47]. Growth cones steer towards a source of attractive guidance molecules, such as netrins and brain-derived neurotrophic factor (BDNF), and steer away from the source of repulsive guidance factors, such as semaphorins and slits [44,45]. Inhibition of protein synthesis suppressed growth cone turning induced by a polarized netrin-1 gradient using cell-body-removed axons of *Xenopus* RGCs, indicating that local protein synthesis is required for growth cone turning in response to netrin-1 [16]. To steer a growth cone, it is necessary to supply cytoskeletal proteins to the extended side of the growth cone. A netrin-1 or BDNF gradient promoted change in the distribution of β-actin mRNA and ZBP1 (or Vg1RBP, the *Xenopus* homolog of ZBP1), an RBP for β-actin mRNA, to the near side of growth cones towards the source of attractive guidance factors [48,49]. Netrin-1 and BDNF also induced asymmetric localization of β-actin protein to the near side of growth cones [48,49]. As disruption of the β-actin mRNA–ZBP1 interaction by antisense oligonucleotides blocked asymmetric localization of β-actin protein and growth cone turning [48], asymmetric local protein synthesis of β-actin in growth cones has an important role in growth cone turning. Local translation in growth cones also occurs in response to repulsive factors. Although the asymmetric localization of mRNAs and proteins in growth cones remains unclear, semaphorin-3A (Sema3A) and slit-2 elicit local translation of RhoA and cofilin mRNAs, respectively, in growth cones [50,51]. As cofilin is an actin-depolymerizing factor and RhoA is involved in actin polymerization/depolymerization, repulsive factors promote local translation of proteins related to reorganization of actin filaments. Considering that local translation of β-actin is induced by attractive factors, different extracellular factors induce local translation from different subsets of mRNAs in growth cones according to attractive/repulsive responses. 

To express different sets of mRNAs in response to different axon guidance factors, it is necessary that different intracellular signaling pathways operate from specific receptors to the RBPs bound to specific mRNAs. A phosphorylation-deficient mutant of ZBP1 at Tyr396 inhibited local translation of β-actin in growth cones induced by BDNF, and suppressed growth cone turning in response to a gradient of attractive guidance factors BDNF and netrin-1 [52,53]. Because phosphorylation of ZBP1 at Tyr396 by Src family kinases releases β-actin mRNA and elicits translation of β-actin [54], these findings suggest that the netrin receptor DCC (deleted in colorectal cancer) and the BDNF receptor TrkB activate Src family kinases to induce phosphorylation of ZBP1, thus initiating translation. Although it is unclear whether ZBP1-containing granules are formed by phase separation, this mechanism involving PTMs of RBPs confers specificity for translation from the transcriptome of growth cones in response to specific extracellular factors. Another example of PTMs of RBPs is FMRP, a causative gene product of Fragile X syndrome, which is a neurodevelopmental disease that causes intellectual disability and autism spectrum disorder (ASD). Analysis of mRNAs isolated by crosslinking immunoprecipitation (HITS–CLIP) using an anti-FMRP antibody revealed that FMRP binds 842 kinds of mRNAs, including microtubule associated protein 1B (MAP1B) [55]. FMRP is localized in growth cones of hippocampal neurons and affects growth cone motility [29]. In response to the repulsive factor Sema3A, FMRP mediates local translation of MAP1B and growth cone collapse in a translation-dependent manner [56]. Because dephosphorylation of FMRP by protein phosphatase 2A promotes the release of mRNAs from FMRP to trigger translation [57,58], it is possible that activation of a Sema3A receptor complex composed of plexin A and neuropilin-1 (Nrp1) promotes local translation of specific mRNAs in growth cones via dephosphorylation of FMRP by protein phosphatase 2A. The translational regulation imparted by PTMs of FMRP in growth cones may involve dephosphorylation-induced dispersion of FMRP-containing granules formed by phase separation [59]. These “specific” signaling pathways composed of specific subsets of receptor-RBPs are important to activate translation of specific subsets of mRNAs. Very recently, it was reported that the netrin-1 receptor DCC and Sema3A co-receptor Nrp1 are associated with different RBPs, hnRNP A2/B1 and Staufen1, respectively [60]. Both DCC and Nrp1 also bind ribosomes, possibly to suppress translation until stimulation by guidance factors [60,61]. These findings suggest that different receptors directly interact with distinct RBPs and ribosomes to regulate translation of specific subsets of mRNAs. These direct/indirect connections of distinct RBPs with different receptors reportedly provide a mechanism to translate specific subsets of mRNAs in response to different guidance factors. 

Nascent axonal proteome analysis revealed that different guidance factors induce translation not only from “specific” subsets of mRNAs, but also from “common” subsets [62]. Translational regulation of “common” subsets of mRNAs, which supposedly occurs in both axons and growth cones, is considered to result from activation of “common” translational pathways in growth cones. Rapamycin, an inhibitor of mammalian target of rapamycin (mTOR), suppressed growth cone turning and local translation in growth cones induced by netrin-1 and Sema3A [16,50,63], suggesting that activation of the mTOR pathway promotes translation from mRNA pools inside growth cones. Because growth cones maintain a specific transcriptome different from axons and cell bodies [21], local activation of common translational pathways, such as the mTOR pathway, elicits translation of growth-cone-specific proteins inside growth cones. When gradients of these axon guidance factors are applied to a growth cone, local translation in the stimulated side of the growth cone occurs to generate a gradient of translated growth cone proteins that drive growth cone turning. It is possible that this gradient of newly synthesized proteins promotes growth cone turning regardless of whether the guidance cue is attractive or repulsive. 

Some growth cone responses triggered by extracellular signals, such as axon guidance factors, do not require protein synthesis in axons. For example, lysophosphatidic acid-induced growth cone collapse was not inhibited by protein synthesis inhibitors [16]. Nerve growth factor-induced increases in growth cone filopodia were independent on translation [64]. Interestingly, Sema3A-induced growth cone collapse was protein synthesis-dependent at low but not high concentrations [65,66]. These findings suggest that extracellular signals elicit protein synthesis-dependent and/or -independent intercellular signaling pathways depending on the type and concentration of extracellular signals. This explanation may account for discrepancies in the protein-synthesis dependence of Sema3A-induced growth cone collapse [65,66,67]. 

## 4. Local Translation in Presynapses 

Chemical synapses are composed of presynapses that contain synaptic vesicles (SVs) filled with neurotransmitters, and postsynapses that express receptors for neurotransmitters. As the synaptic clefts between presynapses and postsynapses are only ~20 nm wide, it is difficult to distinguish local translation in presynapses from that in postsynapses. In addition, given that the level of local translation in axons is much lower than that of dendrites (see Introduction), detection of local translation in presynapses may be very difficult. To overcome these difficulties, several state-of-the-art methods have been applied to analyze presynapses, including puromycin-based metabolic labeling to detect actively translating ribosomes, purification of pure synaptosomes composed of the presynaptic cytosol and presynaptic/postsynaptic membrane, and high resolution microscopy techniques such as expansion microscopy and stimulated emission depletion (STED) microscopy [68,69]. 

The existence of ribosomes in presynapses was shown by immuno-gold EM analysis for ribosomal proteins [22,69]. A combination of RiboTag with EM analysis revealed that the ribosomal protein L22 existed in presynapses of RGC axons, which terminate in the superior colliculus of the midbrain [22]. EM analysis using synaptosomes indicated that the ribosomal protein S11 was localized in presynapses [69]. A large majority of presynapses (>75%) contained small (S11) and large (L26) ribosomal proteins, and ribosomal RNA [69]. However, polysomes are difficult to detect in presynapses. Thus, puromycin-based metabolic labeling was used to detect actively translating ribosomes. Active translation was detected in about 40% of presynapses by staining nascent peptides in neurons metabolically labeled with puromycin using EM and expansion microscopy [69]. Although monosomes, which are composed of single ribosomes with mRNA, synthesize only a small number of proteins, it is possible that translation by monosomes is sufficient to supply enough proteins in small subcellular compartments, such as presynapses [68]. Transcriptome analysis of vesicular glutamate transporter 1 (an excitatory presynapse marker)-containing synaptosomes of adult mouse forebrains revealed the enrichment of 468 transcripts compared with the input forebrain transcriptome [69] (Figure 1). Among these enriched transcripts, transcripts of presynaptic active zone proteins were most enriched in the presynaptic transcriptome, indicating specific mRNAs are enriched for local translation in presynapses for their functions. Because this synaptosome preparation contained postsynaptic membrane in addition to presynaptic cytosol and membrane, mRNAs derived from postsynaptic membrane, not only presynaptic mRNAs, were present. However, the particular enriched mRNAs in the synaptosomal transcriptome were included in translatome of RGC axon preparations of the superior colliculus at P0.5, which contained axonal and presynaptic mRNAs but was free from postsynaptic mRNAs (e.g., mRNAs coding β-catenin, kinesin-5C, and ribosomal proteins) [22,69]. These results suggest that specific mRNAs are localized in presynapses for local translation. Several reports have demonstrated localization of RBPs in presynapses [70,71,72] (see below). Collectively, localization of specific mRNAs, actively translating ribosomes (as indicated by puromycin labeling), and RBPs in presynapses indicate local translation of specific proteins in presynapses (Figure 1). 

Local translation within presynapses has been studied in the sea slug *Aplysia* co-culture system, which is composed of sensory presynaptic and motor postsynaptic neurons [73]. After stimulation to induce long-term facilitation, mRNAs for sensorin (a sensory neuron-specific peptide neurotransmitter) rapidly concentrated in the presynapses of sensory neurons with synaptic contact to motor neurons [74]. Live-cell imaging of photoconvertible fluorescent protein translational reporters for sensorin revealed the accumulation of fluorescent proteins in the presynapses of sensory neurons [75], suggesting local translation of sensorin in presynapses during long-term facilitation. With regard to long-term depression, FMRP participates in both pre- and postsynaptic regulation of enduring synaptic plasticity in a translation-dependent manner [76]. These findings suggest the potential involvement of RBPs, such as FMRP, in long-term plasticity (facilitation and depression) via regulation of local translation in presynapses. However, long-term plasticity observed in invertebrate and vertebrate nervous systems exhibit differences in translation-dependency. Although long-term plasticity in invertebrate models is largely dependent on presynaptic translation, late phase LTP, which is the most studied form of long-term plasticity in mammals, is largely dependent on postsynaptic translation [2,77]. To investigate local translation in presynapses of mammalian neurons, some researchers have recently focused on local translation in presynapses during synaptogenesis. 

Several presynaptic proteins have been identified as proteins synthesized in presynapses during synaptogenesis. β-catenin was locally synthesized during presynapse formation artificially induced by poly-D-lysine beads in hippocampal culture [78]. Synaptosome-associated protein 25 (SNAP25), a t-SNARE protein in the active zone, was also locally translated in presynapses formed by poly-D-lysine beads [79]. Recently, beads coated with leucine-rich repeat transmembrane neuronal 2 (LRRTM2), an excitatory presynaptic organizer protein, were found to promote local translation of the active zone protein Munc18-1 during presynapse formation of cortical neurons [72]. HITS-CLIP analysis revealed that FMRP binds to *Stxbp1* (encodes Munc18-1) mRNA [55]. In neurons with knockout of *Fmr1* (the gene coding FMRP, *Fmr1*-KO), local translation of Munc18-1 in presynapses was increased 1.5-fold compared with wild-type neurons [72]. These findings suggest the potential role of FMRP as a translational suppressor in local translation of Munc18-1. Localization of FMRP in presynapses has been investigated using both in vitro and in vivo systems. FMRP accumulates as granules in presynapses formed artificially by LRRTM2 beads [72]. In vivo, FMRP is localized as granules in presynapses of olfactory sensory neurons and hippocampal neurons, as visualized by EM [71]. FMRP-containing granules (FXGs, also known as fragile X granules) in presynapses contain ribosomal RNA and proteins, as well as other FMRP family proteins (such as FXR1p and FXR2p) [71,80,81]. FXGs also contain some specific mRNAs (e.g., β-catenin and MAP1B) [80,81], suggesting regulation of local translation for mRNAs of FMRP targets. As FMRP binds mRNAs encoding about 30% of proteins in the presynaptic proteome [55], FMRP is considered a key RBP for regulating local protein synthesis of presynaptic proteins. In fact, proteomics analysis of synaptosomes from *Fmr-*KO cortices revealed upregulated expression levels of more than ten presynaptic proteins, including Munc18-1, SNAP25, and Rab3-interacting molecule (RIM), compared with wild-type cortices, suggesting the possible involvement of FMRP in translation of these presynaptic proteins [82]. With the exception of FMRP family proteins, the presence of RBPs in presynapses has rarely been reported. STED microscopy, a super-resolution microscopy, revealed that localization of the RNA/DNA binding protein FUS in presynapses [70]. FUS reportedly interacts with the tandem Agenet domain of FMRP [83]. These findings suggest the possibility that several RBPs form complexes/granules to regulate local translation in presynapses using the presynaptic transcriptome. 

Mechanisms for regulating local translation of the presynaptic transcriptome during synaptogenesis are largely unknown. Neurexin is a presynaptic transmembrane protein that binds to LRRTM2, neuroligin-1, and Cbln1 to induce presynapse formation [84,85]. Stimulation of neurexin promotes binding of the intracellular domain of neurexin to CASK to form active zones [84]. However, it remains unclear how binding to CASK activates signaling pathways for local translation, regardless of “specific” or “common” pathways. It is also completely unknown whether neurexin binds ribosomes, specific RBPs, or subsets of mRNAs. Thus, further research is necessary to clarify the mechanism for translation stimulated by presynaptic organizer proteins. 

## 5. Sites for Translation in Growth Cones and Presynapses

**Figure 2:**
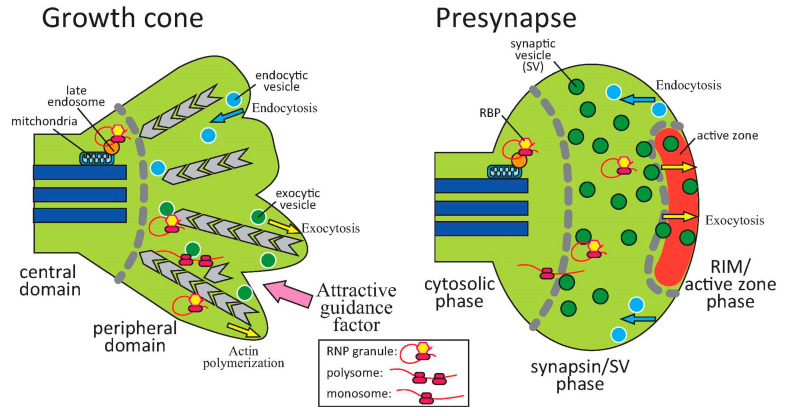
Sites of local translation in growth cones and presynapses. Growth cones are composed of two domains: central domains and peripheral domains. Late endosomes in proximity to mitochondria serve as platforms to bind RNP granules for local translation, which also occurs in the central domain of growth cones. In response to attractive guidance factors, RNP granules localize on the stimulated side, followed by local translation. Presynapses are separated into three phases by phase separation: cytosolic, synapsin/synaptic vesicle (SV), and Rab3-interacting molecule (RIM)/active zone phases. RNP granules (FMRP-containing granules) often localize at the synapsin/SV phase. Ribosomes are sometimes at the synapsin/SV phase; however, they are offset from the active zone.

During transport along axons to growth cones and presynapses, RBPs and mRNAs form neuronal RNP granules (transport granules) as membraneless organelles by phase separation. This granule formation results in suppression of translation until receptors receive extracellular signals to promote local translation. After the granules reach their destinations, sites of translation inside growth cones and presynapses are unknown. Recent studies suggest that late endosomes in proximity to mitochondria serve as platforms to bind RBPs with mRNAs and translate mRNAs to proteins [86] (Figure 2). As membraneless organelles, RNP granules are reportedly tethered to late endosomes and lysosomes, which are membrane-bound organelles, possibly via adaptor proteins such as annexin A11 [87]; thus, it is possible that these membrane-bound organelles associate with RNP granules to serve as platforms for local translation [88]. In the case of growth cones, late endosomes and mitochondria localize near the central domains where axon microtubule rails end, and rarely at the peripheral domain (lamellipodia and filopodia). Because ZBP1, β-actin mRNAs, and β-actin proteins predominantly localize in the central domains, local translation of β-actin is considered to mainly occur in the central domain. However, when growth cones were stimulated by attractive guidance factors netrin-1 and BDNF, localization of ZBP1, β-actin mRNAs, and β-actin proteins changed to the lamellipodia and filopodia of the stimulated side of growth cones [48,89] (Figure 2). Single molecule translation imaging revealed that the β-actin translation induced by netrin-1 occurs at a higher level in the peripheral region of growth cones [90]. Thus, local translation of β-actin is considered to occur in lamellipodia and filopodia, which are peripheral parts of growth cones in response to guidance factors. Indeed, EM analysis demonstrated that ribosomes are localized underneath the plasma membrane of growth cone lamellipodia [60]. 

It is unknown how local translation operates in the peripheral parts of growth cones, where mitochondria and late endosomes are scarcely localized. One possibility is that exocytic vesicles function as platforms for local translation (Figure 2). Attractive guidance factors promote exocytosis to the stimulated side of growth cones [91,92]. Therefore, it is possible that RNP granules hitchhike from late endosomes and lysosomes to exocytic vesicles, followed by local translation. As repulsive factors promote endocytosis on the stimulated side of growth cones [92], lysosomes accumulate on the stimulated side to serve as platforms for translation and localization of guidance factor receptors, such as Nrp1, which bind RBPs. Another possibility is that RNP granules, such as ZBP1-containing granules, are delivered to the peripheral domain of growth cones after departure from the central domain by myosin Va [93], a motor protein on the actin filament (Figure 2). Future work is necessary to examine co-localization of RBPs and mRNAs with exocytic vesicles and lysosomes, and to identify sites of translation (e.g., using puromycin) during growth cone turning.

Unlike growth cones composed of central and peripheral domains, presynapses consist of three parts: the active zone, SV pools (readily releasable, recycling, and reserve pools), and other cytosolic space, including mitochondria. FXGs, which are FMRP-containing RNP granules, are often localized at SV pools [71] (Figure 2). The ribosomal protein RPS11 was also found at SV pools, but was offset from the active zone [69]. These findings suggest that local protein synthesis occurs at the cytosolic spaces and SV pools, but not at the active zone. At synaptic pools, SVs may serve as platforms for local translation at presynapses, such as exocytic vesicles of growth cones. Another possibility is that RNP granules are transported from the roots of presynapses, where microtubules and mitochondria exist, to SV pools for local translation by myosin Va or other motor proteins. Recent reports indicate that the SV protein synapsin 1 condenses into liquid droplets, suggesting that phase separation of synapsin 1 promotes clustering of SVs at presynapses [94]. Furthermore, the active zone proteins RIM and RIM-binding protein (RIM-BP) form condensates via phase separation to generate clusters of Ca^2+^ channels [95]. These findings led to the novel idea that presynapses are separated into three phases: the RIM/active zone, synapsin/SV, and other cytosolic (liquid) phases [31,96] (Figure 2). Because FXGs localize at SV pools, it is possible that FMRP suppresses local translation to maintain mRNAs and translational machinery at the synapsin/SV phase. Once signals to initiate translation are received for presynapse formation/synaptic plasticity, FMRP is dephosphorylated and FXGs, which are formed by phase-separation, are considered to be dispersed to initiate translation [59]. The surrounding phase environment (synapsin/SV phase) may affect the process of forming/dispersing FXGs by phase separation. However, further studies to detect translating ribosomes and FXGs in presynapses in response to extracellular signals at super resolution are necessary. 

## 6. Pathophysiological Role of Local Translation in Growth Cones and Presynapses

Because dysfunction of local translation impacts various axonal and dendritic functions, dysregulated local translation is implicated in the pathophysiology of neuronal diseases [2,88,97]. However, the involvement of dysregulated local translation inside growth cones and presynapses in neuronal diseases has not been extensively researched owing to the difficulty of detecting defects in local translation within these small subcellular compartments. Nevertheless, recent research progress has revealed the involvement of local translation in growth cones and presynapses in the pathophysiology of several neuronal diseases, including axonal injury, neurodegeneration, and neurodevelopmental diseases. 

Local translation in growth cones plays a critical role in axonal regeneration. After axonal injury, assemblies of new growth cones start to regrow axons [98]. Because local translation of β-actin in growth cones in response to BDNF and neurotrophin-3 is required for axonal extension and attractive growth cone turning [17,52], β-actin translation in growth cones is considered to be important for the regrowth of axons after injury. In fact, blockade of the interaction between β-actin mRNA and ZBP1 using overexpression of the exogenous 3′ untranslated region of β-actin mRNA depleted endogenous β-actin and GAP-43 mRNAs in growth cones and attenuated nerve regeneration after common fibular nerve transection [99]. These results suggest that local translation of β-actin and GAP-43 mRNAs in growth cones promotes nerve regeneration after axotomy. Laminopathies, which are caused by mutations in genes coding for lamin family proteins for nuclear lamina, are a group of genetic disorders including muscular dystrophy, lipodystrophy, and neuropathy. One of type 2 Charcot-Marie-Tooth disease, a neuropathy involving axon degeneration, results from inherited mutations in either mitochondrial proteins or lamin A/C [100], classified as laminopathy. Yoon et al. reported that engrailed-1, a guidance cue protein, promoted local translation of lamin B2 in axons and growth cones, whereas inhibition of lamin B2 translation in axons in vivo resulted in axon degeneration and mitochondrial dysfunction [101]. These results suggest that local translation of lamin B2 plays an important role in axonal survival by regulating mitochondrial function. Thus, it is possible that lamin B2 is also involved in neuropathy including axon degeneration. Local translation in growth cones is also important in the pathophysiology of spinal muscular atrophy (SMA), a neurodegenerative disease that causes progressive weakness of the lower motor neurons [102]. SMA is caused by reduced levels of the survival of motor neuron (SMN) protein, which is involved not only in biogenesis of the spliceosome, but also in localization of RNP granules containing ZBP1 [102]. In SMA-model motor neurons, localization of β-actin and GAP-43 mRNAs, as well as local translation of GAP-43, are reduced in growth cones [103,104]. Because plastin 3, a protective modifier of SMA, is important for increasing F-actin levels during axogenesis and axon outgrowth [105], and extending survival in an SMA model [106], it is possible that increasing local translation of β-actin and GAP-43 in growth cones rescues the SMA phenotype by promoting axogenesis and axon outgrowth. These findings obtained from axotomy and SMA models suggest the potential utility of enhancing local translation of cytoskeletal proteins as a strategy for curing nerve injury and SMA by promoting axonal extension. 

In presynapses, active zone proteins Munc18-1 and SNAP25 are locally translated during artificially induced presynapse formation [72,79]. Munc18-1-deficient mouse neurons exhibited complete loss of neurotransmitter release [107] and SNAP25-deficient neurons did not exhibit evoked release [108], suggesting a critical role in neurotransmitter secretion. Neurons with a heterozygous mutation in the *STXBP1* gene (encoding the Munc18-1 protein), which causes infantile early epileptic encephalopathy (Ohtahara syndrome), exhibited a 30% decrease in the level of Munc18-1 and 50% reduction in neurotransmitter release [109]. Thus, Munc18-1 expression levels may correlate to levels of neurotransmitter release. As expression of Munc18-1 in presynapses was increased 1.5-fold in *Fmr1*-KO neurons [72], the increased level of Munc18-1 resulting from dysregulation of local translation by FMRP may lead to increased neurotransmitter release. In fact, synaptic release from *Fmr1*-KO neurons was increased compared with wild-type neurons [110]. In addition, as the expression level of SNAP25 was also increased in *Fmr1*-KO synaptosomes in the cortex, it is possible that increased neurotransmission resulting from augmented local translation of these active zone proteins is involved in the pathophysiology of fragile X syndrome, a neurodevelopmental disease. Moreover, as mutations of *STXBP1*, *SNAP25*, and *RIMS1* (encoding RIM1) genes have been identified in ASD patients [111,112], local translation of these active zone proteins in presynapses is possibly involved in ASD. 

## 7. Future Perspectives

During the last decade, compelling evidence has accumulated for local translation not only in axons, but also in growth cones and presynapses, largely as a result of recent progress in omics, visualization, and subcellular fractionation techniques. However, local translation in presynapses remains far less understood compared with that in growth cones. Interesting questions for local translation at presynapses are as follows:

1) Is translation by monosomes enough for the demands of presynapses? Because 40–4000 molecules of presynaptic proteins (e.g., 40 molecules of RIM proteins) are predicted to localize within a single presynapse, and one molecule of an average-sized protein is considered to be translated within 1–8 min according to a translation speed of five amino acids/s, local translation by monosomes may be enough to meet the demand of relatively low copy proteins in presynapses [68]. To examine whether this hypothesis is correct, a more sensitive method for detection of translation by monosomes is necessary.
2) Is local translation in presynapses regulated by phase separation? Recent studies using purified proteins in a cell-free system demonstrated that PTMs of FMRP regulate condensate formation and translation by phase separation [59,113]. Considering that presynapses are composed of three phases, it is possible that regulation of phase separation by PTMs of RBPs and the surrounding phase environment play an important role in the formation/dispersion of RNP granules and suppression/initiation of translation. However, it is unclear whether this regulation functions in actual presynapses. To address this, it is necessary to examine whether PTM-deficient mutants of RBPs affect RNP granules in presynapses, and which phases in presynapses are sites of local translation.
3) What are the physiological and pathophysiological roles of local translation in presynapses? Local translation in presynapses is potentially implicated in the formation [72,78,79] and synaptic plasticity [114] of presynapses. Thus, it is also important to investigate the role of presynaptic local translation in neuronal diseases, as described in the previous section. To answer this question, optogenetics-based regulation of translation [115] may be a useful technique to examine the involvement of presynaptic local translation in physiological and pathophysiological conditions.

Answering these questions will lead to new insights into the mechanisms underlying synapse formation and synaptic plasticity, thus opening the door to the discovery of new therapeutic targets for fragile X syndrome, ASD, and other synaptic diseases.
